# Rice Mutants Lacking Starch Synthase I or Branching Enzyme IIb Activity Altered Starch Biosynthetic Protein Complexes

**DOI:** 10.3389/fpls.2018.01817

**Published:** 2018-12-07

**Authors:** Naoko Crofts, Yuriko Iizuka, Natsuko Abe, Satoko Miura, Kana Kikuchi, Ryo Matsushima, Naoko Fujita

**Affiliations:** ^1^Department of Biological Production, Faculty of Bioresource Sciences, Akita Prefectural University, Akita, Japan; ^2^Institute of Plant Science and Resources, Okayama University, Okayama, Japan

**Keywords:** rice, starch, amylopectin, starch synthase, starch branching enzyme, protein–protein interaction

## Abstract

Amylopectin, the major component of starch, is synthesized by synergistic activity of multiple isozymes of starch synthases (SSs) and branching enzymes (BEs). The frequency and length of amylopectin branches determine the functionality of starch. In the rice endosperm, BEIIb generates short side chains of amylopectin and SSI elongates those branches, which can be further elongated by SSIIa. Absence of these enzymes greatly affects amylopectin structure. SSI, SSIIa, and BEIIb associate with each other and with other starch biosynthetic enzymes although SSIIa is low activity in japonica rice. The aim of the current study was to understand how the activity of starch biosynthetic enzyme complexes is compensated in the absence of SSI or BEIIb, and whether the compensatory effects are different in the absence of BEIIb or in the presence of inactive BEIIb. Interactions between starch biosynthetic enzymes were analyzed using one *ss1* null mutant and two *be2b* japonica rice mutants (a mutant producing inactive BEIIb and a mutant that did not produce BEIIb). Soluble proteins extracted from the developing rice seeds were separated by gel filtration chromatography. In the absence of BEIIb activity, BEIIa was eluted in a broad molecular weight range (60–700 kDa). BEIIa in the wild-type was eluted with a mass below 300 kDa. Further, majority of inactive BEIIb co-eluted with SSI, SSIIa, and BEI, in a mass fraction over 700 kDa, whereas only small amounts of these isozymes were found in the wild-type. Compared with the *be2b* lines, the *ss1* mutant showed subtle differences in protein profiles, but the amounts of SSIIa, SSIVb, and BEI in the over-700–kDa fraction were elevated. Immunoprecipitation revealed reduced association of SSIIa and BEIIb in the *ss1* mutant, while the association of BEIIb with SSI, SSIIa, SSIVb, BEI, and BEIIa were more pronounced in the *be2b* mutant that produced inactive BEIIb enzyme. Mass spectrometry and western blotting revealed that SSI, SSIIa, SSIIIa, BEI, BEIIa, starch phosphorylase 1, and pullulanase were bound to the starch granules in the *be2b* mutants, but not in the wild-type and *ss1* mutant. These results will aid the understanding of the mechanism of amylopectin biosynthesis.

## Introduction

Amylopectin, the major component of starch, is composed of highly branched glucose polymers (reviewed by Bertoft, 2017; Goren et al., 2018). It is accumulated in the amyloplasts in storage tissues of plants, e.g., the rice endosperm (reviewed by Goren et al., 2018). The branch length and frequency of amylopectin branching determine the functionality of starch and, ultimately, the application and commercial value of rice grains (Fujita, 2014). Therefore, understanding the molecular mechanisms that govern the amylopectin branch structure is essential.

Amylopectin is synthesized by multiple classes of enzymes. Each class of enzymes contains multiple isozymes with different substrate and product specificities, and with different developmental and tissue-specific expression patterns (Ohdan et al., 2005; Fujita and Nakamura, 2012). According to the current understanding of amylopectin biosynthesis in the rice endosperm, amylopectin is synthesized by a synergistic action of starch synthases (SSs; EC 2.4.1.21), which elongate α-1,4–linked glucans using ADP-glucose as a substrate, and starch branching enzymes (BEs; EC 2.4.1.18), which generate α-1,6–linked branches (Nakamura et al., 2014). SSIIIa generates long amylopectin chains with a degree of polymerization (DP) >30 (Fujita et al., 2007). BEIIb generates short side chains of amylopectin with DP 6 or 7 (Nishi et al., 2001; Nakamura et al., 2010), and SSI elongates these branches to DP 8–12 (Fujita et al., 2006). The elongated branches can be further elongated by SSIIa (Umemoto et al., 2002; Nakamura et al., 2005; Crofts et al., 2017b). Improperly placed branches are removed by starch debranching enzymes, mainly by isoamylase (ISA; EC 3.2.1.68) (Kubo et al., 1999; Nakamura, 2002; Utsumi et al., 2011), and subsidiarily by pullulanase (PUL; EC 3.2.1.41) (Fujita et al., 2009). Plastidial starch phosphorylase (Pho1; EC 2.4.1.1) elongates α-1,4–linked glucans using glucose-1-phosphate as a substrate. It is thought to be involved in the initiation step of starch biosynthesis in the rice endosperm, although Pho1 can also function to degrade glucan (Satoh et al., 2008). In addition to the above-mentioned starch biosynthetic isozymes, SSIVb (Toyosawa et al., 2016), BEIIa (Satoh et al., 2003; Lee et al., 2017), and the disproportionating enzyme (EC 2.4.1.25) (Dong et al., 2015) are also involved in amylopectin biosynthesis in the rice endosperm.

The SSI and BEIIb are major enzymes that generate short chains of amylopectin in typical japonica rice, since SSIIa from japonica rice possesses only 10% of the activity of SSIIa from typical indica rice (Nakamura et al., 2005). The absence of SSI or BEIIb in japonica rice greatly affects chain length distribution of amylopectin. When SSI is absent, amylopectin branch structure is altered such that DP 6–7 and DP 16–19 fractions increase, and DP 8–12 fraction is reduced (Fujita et al., 2006). When BEIIb activity is absent, regardless of the presence or absence of BEIIb, amylopectin structure is drastically altered such that the number of short chains (DP < 17), particularly DP 8–12, is reduced and the number of long chains is increased (Nishi et al., 2001; Tanaka et al., 2004; Matsushima et al., 2010; Butardo et al., 2011; Nakata et al., 2018). In addition, the absence or reduction of BEIIb changes the crystallinity of starch, increases its gelatinization temperature, and lowers solubility in alkaline solutions (Nishi et al., 2001; Tanaka et al., 2004; Matsushima et al., 2010; Butardo et al., 2011; Nakata et al., 2018). Furthermore, the absence of both SSI and BEIIb proteins in japonica rice leads to sterility although a double mutant with reduced SSI levels and lacking BEIIb is able to generate seeds (Abe et al., 2014).

Most of the starch biosynthetic enzymes interact with each other (Crofts et al., 2015, 2017a). One of the best-studied examples is the phosphorylation-dependent formation of the protein complex of SSI, SSIIa, and BEIIb in the amyloplast stroma in the developing endosperms of wheat (Tetlow et al., 2004, 2008) and maize (Hennen-Bierwagen et al., 2008; Liu et al., 2009, 2011, 2012; Makhmoudova et al., 2014). This trimeric protein complex of approximately 230 kDa is most likely important for protein binding to starch granules and for the synthesis of amylopectin clusters (Crofts et al., 2017a) and is commonly found in cereals, including barley (Ahmed et al., 2015) and rice (Nakamura et al., 2014; Crofts et al., 2015). Although SSIIa in wild-type japonica rice possesses only 10% activity of that found in typical indica rice (Nakamura et al., 2005), SSIIa in the developing japonica rice endosperm associates with SSI, BEIIb, and other starch biosynthetic enzymes to form protein complexes of a wide molecular weight range (200–700 kDa) (Crofts et al., 2015; Hayashi et al., 2018; Miura et al., 2018). In addition to the trimeric protein complexes, protein–protein interactions also occur between BEs and PUL (Crofts et al., 2015), BEs and Pho1 (Crofts et al., 2015; Nakamura et al., 2017), Pho1 and the disproportionating enzyme (Hwang et al., 2016), and ISA and a carbohydrate-binding module 48 domain-containing protein (Peng et al., 2014) in the developing rice seeds. Furthermore, SSIIIa in the developing maize kernels associates with multiple starch biosynthetic enzymes, such as BEs, pyruvate phosphate dikinase (PPDK), and ADP-glucose pyrophosphorylase (Hennen-Bierwagen et al., 2009).

Although the exact function of each isozyme cannot be complemented by other isozymes, the formation of complexes of starch biosynthetic enzymes is compensated by isozymes when the expression levels of specific enzymes are altered (Liu et al., 2009, 2011, 2012; Hayashi et al., 2018; Miura et al., 2018). The physicochemical properties of starch from SSI and BEIIb mutant rice lines have been well studied; however, it is not known whether the interactions of starch biosynthetic enzymes in these rice mutants are altered. Therefore, analyses of protein complex formation of starch biosynthetic enzymes using mutants that lack specific enzymes will enable the understanding of the relationship between the interactions of starch biosynthetic enzymes and amylopectin structure. Protein complex formation in the *be2b* mutants has been studied in maize. However, in the current study, we describe the different effects of the absence of BEIIb or the presence of BEIIb inactivated by a substitution in a domain different from that in the previously analyzed maize mutants.

In the current study, to understand how the composition of starch biosynthetic enzyme complexes is compensated in the absence of SSI or BEIIb in japonica rice, soluble proteins extracted from the developing rice seeds of the *ss1* or *be2b* mutant rice lines (lacking BEIIb or producing inactive BEIIb) were analyzed by gel-filtration chromatography and co-immunoprecipitation. In addition, to understand the relationship between the complex formation of starch biosynthetic proteins and the affinity of these enzymes for the starch granule, starch granule-bound proteins were identified by nano-liquid chromatography-tandem mass spectrometry (nano-LC-MS/MS). Finally, the possible compensatory effects on starch biosynthetic protein complex formation are discussed.

## Materials and Methods

### Plant Material

Japonica rice mutant line *e7*, an SSI-deficient mutant (*ss1*), was generated by insertion of retrotransposon *Tos 17* in exon 7 of the *SSI* gene in the parental line Nipponbare (Nip), as described previously (Fujita et al., 2006). Nip was used as a control for the *ss1* mutant. Line *EM10* [*be2b* (-)], which lacks BEIIb protein, was previously isolated from *N*-nitroso-*N*-methylurea mutant population of the japonica cultivar, Kinmaze (Kin) (Nishi et al., 2001). Kin was used as a control for *EM10* [*be2b* (-)] mutant. Line #*1411* [*be2b* (+)] was generated by backcrossing twice a high-yield, large grain rice cultivar Akita 63 (Akt) (Mae et al., 2006) with the *ssg3* japonica rice mutant, which produces inactive BEIIb protein that harbors Gly645Arg substitution (Matsushima et al., 2010). Although expression levels of BEIIb protein in *ssg3* is identical to the wild-type, the amylopectin chain length distribution pattern is identical to *EM10* showing BEIIb in *ssg3* is inactive (Matsushima et al., 2010). Akt was used as a control for line #*1411*. All rice plants were grown in an experimental paddy field of the Akita Prefectural University under natural conditions during the summer months (April to October). Mid-developing seeds (10–15 days after flowering) harvested from 20 plants per line were collected and stored at -30°C until use.

### Chain Length Distribution Analyses of Amylopectin

Starch from mature rice grain was solubilized by alkaline and debranching of amylopectin was performed by *Pseudomonas* isoamylase (generous gift from Hayashibara Co., Ltd.) as described previously (Fujita et al., 2001). Debranched glucans were labeled by 1-aminopyrine-3,6,8-trisulfonic acid, and analyzed by capillary electrophoresis (P/ACE MDQ carbohydrate system, AB Sciex) according to the methods described (O’Shea and Morell, 1996; Fujita et al., 2001).

### Gel Filtration Chromatography

Soluble proteins were extracted from 700 mg of de-hulled developing seeds using 1.5 volumes of a buffer containing 10 mM Hepes-KOH (pH 7.5), 100 mM NaCl, and 10 μL/mL protease inhibitor (Sigma), as described previously (Crofts et al., 2015). After centrifugation, supernatants were filtered through a 0.45-μm syringe filter and 500 μL was loaded onto Superdex 200 gel filtration chromatography column (Φ16 mm × 410 mm) connected to an AKTAprime plus chromatography system (GE Healthcare), at 1 mL/min with 10 mM Hepes-KOH (pH 7.5), 100 mM NaCl. Then, 2-mL fractions were collected and concentrated to 80 μL using Amicon Ultra cartridges with 30-kDa cut-off (Merck). Concentrated fractions were supplemented with 40 μL of 3× native polyacrylamide gel electrophoresis (PAGE) sample buffer containing 0.625 M Tris-HCl (pH 7.0), 0.2% bromophenol blue, and 50% glycerol, or 3× sodium dodecyl sulfate (SDS) PAGE sample buffer containing 0.1 M Tris-HCl (pH 6.8), 10% SDS, 12% β-mercaptoethanol, 0.2% bromophenol blue, and 20% glycerol. Gel filtration experiments were performed at least twice and reproducible results were obtained. One representative result of western blot or native-PAGE gel is shown.

### Co-immunoprecipitation

Soluble proteins, extracted as described above, were incubated with isozyme-specific antibodies and protein A sepharose (Sigma), as described elsewhere (Crofts et al., 2015). After extensive washing, protein complexes were eluted by boiling in SDS-PAGE sample buffer. 5 μL each was used for western blotting. Experiments were performed at least twice and reproducible results were obtained. One representative result of western blot is shown.

### Western Blotting

Samples were loaded onto 7.5% acrylamide SDS-PAGE gels. After blotting onto a polyvinylidene fluoride membrane, the membranes were incubated with isozyme-specific antisera as the primary antibodies, and the remaining procedures were performed as described previously (Crofts et al., 2015).

### Native-PAGE Activity Staining

Gel filtration fraction (7.5 μL), prepared as described above, were resolved in native-PAGE gels containing 0.0001% oyster glycogen as a primer. The experiment was performed as previously reported (Yamanouchi and Nakamura, 1992).

### Isolation and Identification of Starch Granule-Bound Proteins

Ten grains of the developing rice seeds were ground by plastic pestle. Then, soluble proteins and proteins loosely bound to the starch granules were removed by extracting three times in 1 mL of a buffer containing 0.125 M Tris-HCl (pH 6.8), 4% SDS, and 5% β-mercaptoethanol. After centrifugation, the starch pellet was washed twice with 1 mL of water and twice with 1 mL of acetone. Starch was then dried and the starch granule-bound proteins were extracted using 20 volumes (w/v) of denaturing buffer containing 8 M urea, 0.125 M Tris-HCl (pH 6.8), 4% SDS, and 5% β-mercaptoethanol, at 37°C for 2 h with agitation. After centrifugation, the supernatant was loaded on 7.5% SDS-PAGE gels. The gels were silver-stained and photographed, or used for western blotting. Duplicated gels were stained by Coomassie brilliant blue and the bands were excised.

The gel pieces were treated with trypsin and analyzed by nano-LC-MS/MS, QSTAR XL (Applied Biosystems) connected with Bio NanoLC (HiQ sil C18W-3, Φ0.1 mm × 50 mm; KYA TECH), by standard protocol at JBioS Co., Ltd. The data generated were submitted to search with the Mascot (Matrix-Science, London, United Kingdom) algorithm. The protein identification was carried out using *Oryza sativa* NCBInr 20110709 protein sequence database. The Mascot search parameters were as follows: one maximum missed cleavages, mass tolerance of ±0.2 Da, cysteine carbamidomethylation (variable modification), methionine oxidation (variable modification), and charge state of +2 and +3. Only those proteins matched with scores at a 95% confidence level were considered as significant hits.

## Results

### Amylopectin Structure of the *ss1* and *be2b* Mutants

**FIGURE 1:**
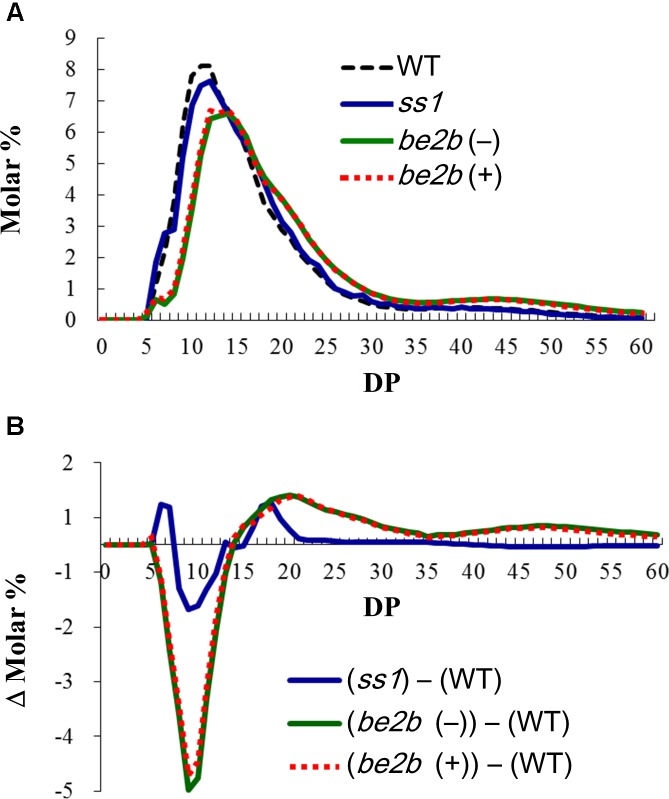
Amylopectin structure of the *ss1* null mutant and two *be2b* mutants analyzed by capillary electrophoresis following debranching the endosperm starch with isoamylase. **(A)** Chain length distribution patterns of WT (Nip) in black dotted line, *ss1* null mutant in blue, and *be2b* mutants; (–), *EM10* in green, which lacks BEIIb protein; (+), *#1411* in red dotted line, which produces inactive BEIIb protein. **(B)** The detailed differences of the chain length distribution patterns shown as Δ molar % by subtracting the data of WT (Nip) from that of the *ss1* or *be2b* mutant lines.

Amylopectin structure of the *ss1* null mutant and two *be2b* mutants lacking BEIIb activity (lines #*1411* and *EM10*) were analyzed by capillary electrophoresis following debranching the endosperm starch with isoamylase (Figure 1A). The detailed differences of the chain length distribution patterns are shown as Δ molar % by subtracting the data of WT (Nip) from that of the *ss1* or *be2b* mutant lines (Figure 1B). The results from one of the representatives are shown (Figure 1). The chain length distribution pattern of all WT lines (Nip, Kin, and Akt) were identical (data not shown), and the data from Nip was used as WT (Figure 1).

The results of *ss1* null mutant showed the typical chain length distribution pattern of *ss1* mutants (Fujita et al., 2006), and that an increase in the proportion of chains of DP 6–7 and 16–20, and a reduction in the proportion of glucan chains of DP 8–12 were observed (Figures 1A,B).

Regard less of the presence or absence of inactive BEIIb protein, both *be2b* mutants showed a similar chain length distribution pattern (Figure 1A). A drastic decrease in the proportion of short chains (DP 6–13) and an increase in the proportion of longer glucan chains (>DP 14) were observed. These chain length distribution patterns of both *be2b* mutants were typical of that found in previous studies (Nishi et al., 2001; Matsushima et al., 2010; Liu et al., 2011). A small difference among two *be2b* mutant lines at DP 9–10 were likely due to environmental factor as the seeds from different harvest years showed no differences (data not shown).

### Molecular Weight Distribution of Starch Biosynthetic Enzymes in the *ss1* Mutant

**FIGURE 2:**
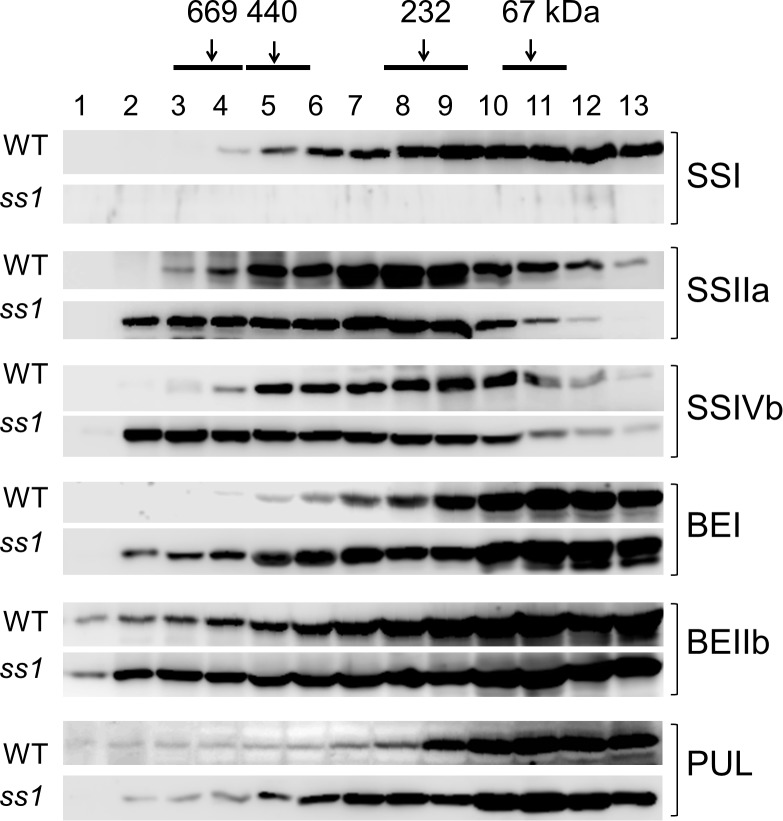
Altered molecular weight distributions of starch biosynthetic enzymes in the *ss1* mutant and its parental line, Nip (WT). Fractions 1–13 obtained after gel filtration chromatography of soluble proteins extracted from the developing seeds were analyzed by western blotting with specific antibodies. Top, numbers (kDa) indicate molecular weight standards. The images are representative of three.

Soluble proteins extracted from the developing seeds of the *ss1* null mutant were separated by gel filtration chromatography. The eluted fractions were denatured, and analyzed by western blotting using isozyme-specific antibodies (Figure 2). The elution patterns of major starch biosynthetic enzymes were compared with those of the parental wild-type, Nip. As expected, SSI was absent from the fractions of the *ss1* mutant. The amounts of SSIIa and SSIVb in fractions 2–4 (>700 kDa) were higher than in the wild-type, and in fraction 7–13 (<300 kDa) were lower than in the wild-type (Figure 2). The elution pattern of SSIIIa was similar to that of the wild-type (Supplementary Figure S1). Although the elution patterns of BEIIa, ISA1, and Pho1 were unchanged (Supplementary Figure S1), the amounts of BEI and BEIIb in fractions 2–8 (>300 kDa) and PUL in fractions 5–8 (200–400 kDa) were elevated (Figure 2). Native-PAGE activity staining of the SS isozymes revealed a protein pattern similar to that obtained by western blotting (data not shown).

### Molecular Weight Distribution of Starch Biosynthetic Enzymes in the *be2b* Mutants

**FIGURE 3:**
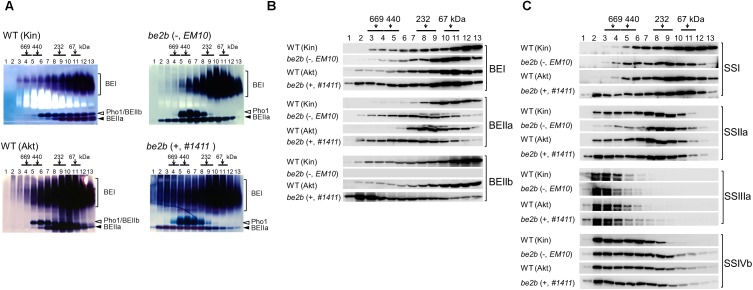
Altered molecular weight distributions of starch biosynthetic enzymes from the *be2b* mutants lacking or producing inactive BEIIb protein, and their parental lines. Fractions 1–13 obtained after gel filtration chromatography of soluble proteins extracted from the developing seeds were analyzed by native-PAGE activity staining **(A)**. The fractions were denatured and used for western blotting with BEI, BEIIa, and BEIIb antibodies **(B)**, and western blotting with SSI, SSIIa, SSIIIa, and SSIVb antibodies **(C)**. Top, numbers (kDa) indicate molecular weight standards; (–), line *EM10*, which lacks BEIIb protein; (+), line *#1411*, which produces inactive BEIIb protein. The parental wild-type (WT) of line *EM10* is Kin and that of line *#1411* is Akt. The images are representative of three.

Soluble proteins were extracted from the developing rice seeds of two *be2b* mutants lacking BEIIb activity (lines #*1411* and *EM10*). Soluble proteins obtained from lines #*1411* and *EM10*, and the parental wild-types Akt and Kin (respectively) were separated by gel filtration chromatography. The eluted fractions were analyzed by native-PAGE activity staining (Figure 3A); denatured proteins were analyzed by western blotting with isozyme-specific antibodies (Figures 3B,C and Supplementary Figure S2). Although some differences in elution patterns of the SS isozymes in wild-type and mutant lines were noted (Figure 3C), striking differences were observed for the BE isozymes (Figure 2).

BEIIb activity was absent in both *be2b* lines (Figure 3A), and BEIIb protein was absent in line *EM10* fractions, as expected (Figure 3B). By contrast, most of the inactive BEIIb protein in line #*1411* was eluted in high-molecular-weight fractions (>700 kDa) enriched in fractions 2 and 3; only a small amount of BEIIb was found in fractions 2 and 3 from the wild-type (Figure 3B). Less BEIIb eluted in a fraction corresponding to its monomeric molecular weight (fractions 10–13) in line #*1411* than in the wild-type (Figure 3B). In addition, in the absence of BEIIb activity, regardless of the presence of the inactive BEIIb protein, active BEIIa was detected in the high-molecular-weight fractions (fractions 2–5; >400 kDa) in lines *EM10* and #*1411*; neither BEIIa protein nor its activity were apparent in these fractions in the wild-type (Figures 3A,B). Further, the elution pattern of BEI was different in the *be2b* mutant lines (Figure 3B). The elution pattern of BEI in line *EM10* was relatively similar to that of the wild-type, with slight reduction of the amount of BEI in fractions 3–6 and a slight increase in fractions 9–11 compared to the wild-type (Kin). However, the amount of BEI that eluted in fractions 1–7 was considerably elevated in line #*1411* (Figure 3B). The amounts of BEI and BEIIa proteins detected by western blotting (Figure 3B) correlated with the strength of BEI and BEIIa activities in each fraction in all rice lines analyzed (Figure 3A).

The elution pattern of SSI was different in the *be2b* mutant lines. The amount of SSI in fractions 4 and 5 was elevated, and that in fractions 12 and 13 was reduced in line *EM10* compared with the wild-type (Kin), while more SSI eluted in fractions 2–4 in line #*1411* than in the wild-type (Akt; Figure 3C). Similarly, the amounts of SSIIa in line *EM10* fractions 2–6 were lower than in the wild-type (Kin), while more SSIIa eluted in line #*1411* fractions 2 and 3 than in the wild-type (Akt; Figure 3C). The elution patterns of SSIIIa and SSIVb in all analyzed rice lines were similar (Figure 3C). The activities of detectable SS isozymes in each fraction in all rice lines analyzed (data not shown) correlated with the amount of each isozyme indicated by western blotting (Figure 3C).

The elution patterns of ISA1 in both *be2b* mutant lines were almost identical, while the amount of PUL in fractions 1 and 2 was slightly elevated in line #*1411* (Supplementary Figure S2). Native-PAGE activity staining (Figure 3A) and western blotting (Supplementary Figure S2) of Pho1 indicated that the activity and amounts of Pho1 in both *be2b* mutant lines were slightly higher than in the respective wild-type lines.

### Protein Associations of Starch Biosynthetic Enzymes in the *ss1* and *be2b* Mutants

**FIGURE 4:**
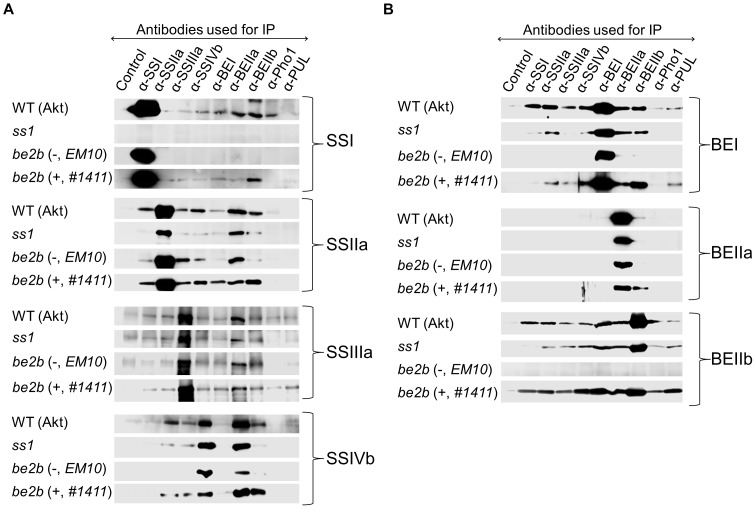
Co-immunoprecipitation of proteins from the *ss1* and *be2b* mutants. (–), line *EM10*, which lacks BEIIb protein; (+), line *#1411*, which produces inactive BEIIb protein. The wild-type (WT) is Akt. Soluble proteins extracted from developing seeds were incubated with the indicated isozyme-specific antibodies and protein A sepharose. After washing, the captured proteins were analyzed by western blotting. Control experiment was performed using pre-immune antisera. **(A)** Western blotting of SS isozymes. **(B)** Western blotting of BE isozymes. The images are representative of at least three.

To investigate the differences in the composition of protein complexes in starch biosynthetic enzymes in the *ss1* and *be2b* mutant rice lines, co-immunoprecipitation experiments were performed using isozyme-specific antisera, and soluble proteins were extracted from the developing seeds of the mutants and their parental lines (Figures 4A,B and Supplementary Figures S3A,B). Control experiments were performed using pre-immunization sera. Each antibody recognized and immunoprecipitated the respective antigen. As expected, neither SSI nor BEIIb were captured in the *ss1* mutant or line *EM10*, respectively. The results of co-immunoprecipitation for the three wild-type lines were almost identical (Figure 4 and Supplementary Figure S3).

Strong pairwise associations detected by reciprocal co-immunoprecipitation were observed for SSI–BEIIb, SSIIa–BEIIb, BEI–BEIIb, and BEIIa–BEIIb in wild-type plants. Clear western blot signals were obtained for only one way in the co-immunoprecipitation experiments in some cases; of such interactions, BEIIa–SSIIa, BEIIa–SSIIIa, BEIIa–SSIVb, and BEIIa–Pho1 were commonly observed in all lines analyzed (first name, antibody used for immunoprecipitation; second name, isozyme subsequently detected by western blotting) (Figures 4A,B and Supplementary Figures S3A,B).

In the *ss1* mutant, interactions of SSIIa with other starch biosynthetic enzymes were reduced and that between SSIIa and BEIIb was reduced, as assessed by reciprocal co-immunoprecipitations; however, the yield of SSIIa protein by SSIIa immunoprecipitation was also reduced (Figures 4A,B) although the amount of SSIIa in the soluble fraction was similar to that in other lines (Figure 2). In line *EM10*, the interactions of SSI and BEI with other starch biosynthetic enzymes, such as SSI–SSIIa, SSI–BEI, SSIIa–BEI, and BEIIa–BEI, were reduced compared to the wild-type (Figures 4A,B). By contrast, in line #*1411*, the interactions between SSI–BEIIb, SSIIa–BEIIb, SSIVb–BEIIb, BEI–BEIIb, and BEIIa–BEIIb, as observed by reciprocal co-immunoprecipitation, were stronger than in the wild-type. The PUL–BEIIb interaction was also more pronounced in line #*1411* than in the wild-type. These observations supported the observed co-elution of SSI, SSIIa, SSIIIa, SSIVb, BEI, BEIIa, and PUL with inactive BEIIb in the high-molecular weight fraction (>700 kDa) in line #*1411* (Figure 3).

### Identification of Starch Granule-Bound Proteins

**FIGURE 5:**
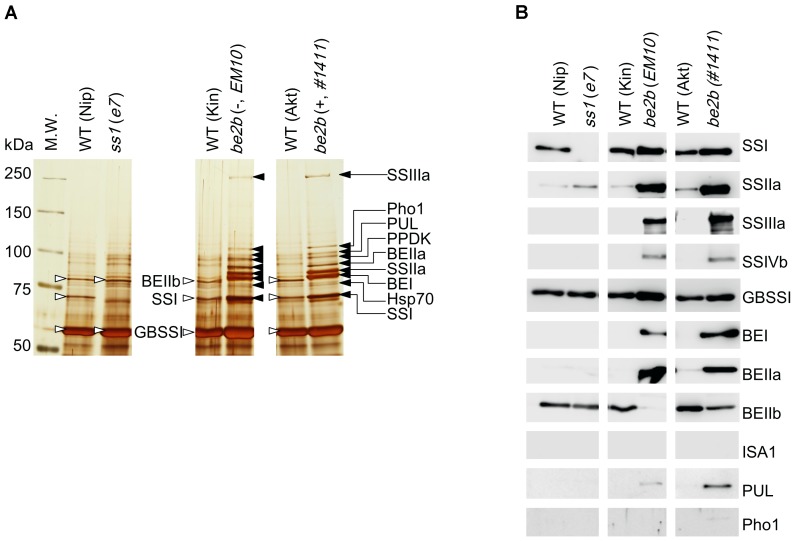
Starch granule-bound proteins isolated from the developing rice seeds of the *ss1* and *be2b* mutants, and their parental lines. **(A)** Silver stained gel. **(B)** Western blotting with the indicated antibodies. Black arrows indicate proteins identified by nano-LC-MS/MS in line *EM10*, which were also found in line *#1411* by western blotting. White arrowheads indicate proteins commonly found in the wild-types Nip, Kin, and Akt, and shown to be BEIIb, SSI, and GBSSI by western blotting with isozyme-specific antibodies. The images are representative of three. All of the lines were analyzed in the same membrane along with other lines omitted from this study, which were cropped out from the final figure.

To investigate the relationship between the formation of a protein complex of starch biosynthetic enzymes and the affinity of those enzymes to the starch granule, starch granule-bound proteins were analyzed. Starch granule-bound proteins were extracted from all mutant and wild-type strains. The proteins were resolved by SDS-PAGE and silver stained (Figure 5A), and also analyzed by western blotting (Figure 5B). The starch granule-bound proteins isolated from line *EM10* were loaded on a replicate SDS-PAGE gel, and protein bands were excised and analyzed by nano-LC-MS/MS (Table 1 and Supplementary Figures S4A–I).

**Table 1:** Starch granule-bound proteins from the *be2b* mutant rice line *EM10* identified by nano-LC-MS/MS.

Protein	M_r_^a^	Score^b^	Peptide^c^	Coverage^d^	UniProt accession no.^e^	GI^f^
SSIIIa	201	358	41	25	Q6Z1D6	40253646
Pho1	109	475	37	37	B3IYE3	190689248
PUL	100	785	56	60	B9FDM0	222628355
PPDK 1	103	644	25	21	Q6AVA8	218196777
BEIIa	95	191	30	35	Q9SXI9	5689138
SSIIa	88	1112	92	55	Q0DDE3	60417785
BEI	93	529	50	48	Q01401	218149
70-kDa heat shock protein	74	1041	32	48	Q2QV45	218186646
SSI	71	507	49	48	Q0DEC8	122168579


*^a^Molecular weight of each protein predicted by Mascot (kDa). ^b^Mascot score. ^c^Number of peptides identified. ^d^%Amino acid sequence coverage of the identified peptides. ^e^Accession number at the European Bioinformatics Institute (EMBL-EBI; https://www.uniprot.org/). ^f^Accession number at the National Center for Biotechnology Information (NCBI; https://www.ncbi.nlm.nih.gov/).*

As determined by western blotting, three major proteins, likely to be BEIIb, SSI, and GBSSI, were detected in all wild-type plants (Nip, Kin, and Akt). As expected, SSI was not detected in the *ss1* mutant, but the amount of SSIIa in starch granule-bound proteins from this strain was slightly higher than in the wild-type (Figures 5A,B). By contrast, the amounts of starch granule-bound proteins detected in both *be2b* mutant lines were typically higher than those in the wild-types (Figures 5A,B). SSI, SSIIa, SSIIIa, GBSSI, BEI, and BEIIa were starch granule-bound proteins strongly detected by western blotting in the *be2b* mutant lines; SSIVb and PUL were only weakly detected (Figures 5A,B). The only difference in the starch granule-bound proteins in lines *EM10* and *#1411* was the presence of BEIIb protein in line *#1411* (Figures 5A,B). The amount of BEIIb detected in line *#1411* was noticeably lower than that in the parental wild-type (Figure 5B).

Starch granule-bound proteins from line *EM10* were identified by nano-LC-MS/MS as SSIIIa, Pho1, PUL, PPDK, BEIIa, SSIIa, BEI, heat shock protein 70, SSI, and GBSSI (Figure 5A, Table 1, and Supplementary Figures S4A–I). Although SSIVb was detected by western blotting of the starch granule-bound proteins from the *be2b* mutants, it was not identified by nano-LC-MS/MS, while Pho1 was identified by nano-LC-MS/MS but not detected by western blotting. These discrepancies may be associated with protein abundance or the sensitivity of methods utilized.

## Discussion

The main objectives of the current study were to investigate the effect of loss of SSI and BEIIb, which play a central role in the production of amylopectin chains within amylopectin clusters, on protein complex formation by starch biosynthetic enzymes in the developing rice seed; to reveal the differences in the compensatory effects of starch biosynthetic proteins in the presence or absence of inactive BEIIb protein; to reveal the differences in the starch granule-bound proteins in the absence of SSI and BEIIb; and to identify starch granule-bound proteins in the *ss1* and *be2b* mutant rice lines.

### Pleiotropic Effects of SSI Loss on the Elution Pattern of Starch Biosynthetic Enzymes in the Developing Rice Seed

**FIGURE 6:**
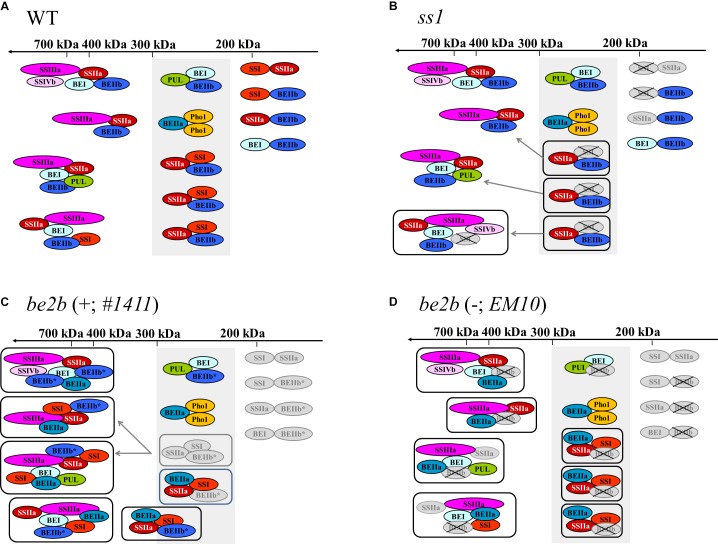
Schematic representation of the proposed starch biosynthetic protein complexes in developing rice seeds. Selected protein complexes expected in the wild-type japonica rice (Crofts et al., 2015; Hayashi et al., 2018) **(A)**, *ss1* mutant **(B)**, *be2b* mutant producing inactive BEIIb **(C)**, and *be2b* mutant lacking BEIIb protein **(D)**. The missing isozymes are crossed-out. Boxes indicate altered protein complex formation. Isozymes whose levels are lower than in the wild-type are indicated in gray. Newly recruited isozymes or isozymes whose levels are higher than in the wild-type are indicated by bold ovals. Gray arrows indicate enzyme recruitment. Shaded box in the 200–300-kDa region indicates the molecular weight range in which the SSI–SSIIa–BEIIb trimeric complex elutes in the wild-type. The asterisk indicates inactive BEIIb. Note that the SSIIa activity in all plant lines used in the current study is low because they have been derived from japonica rice (Nakamura et al., 2005).

The SSI forms a trimeric protein complex (ca. 230 kDa) with SSIIa and BEIIb in the amyloplast stroma in developing wild-type maize kernels (Liu et al., 2012). Similarly, the analysis of soluble proteins suggested the formation of this complex in the developing seed of japonica rice (Crofts et al., 2015). BEI–BEIIb and BEI–PUL associations also take place in the developing seed of japonica rice (Crofts et al., 2015; Hayashi et al., 2018). Some possible protein complexes that form in the wild-type japonica rice are schematically shown by in Figure 6A, although other interactions such as SSI, SSIIa, SSIIIa, BEI, BEIIb, and PUL; SSI, SSIIa, SSIIIa, SSIVa, BEI, BEIIb, and PUL, may occur in wild type rice.

Further, *in vitro* studies demonstrated the functional interaction of recombinant rice SSI and BEIIb enzymes; these interactions simultaneously and synergistically increase the mutual function of the interacting proteins, to generate amylopectin branches in crystalline lamellae (Nakamura et al., 2014).

In the absence of SSIIIa in japonica rice, SSI is found in the high molecular weight fraction (>700 kDa), in which SSIIIa is usually present; by contrast, only a low amount of SSI is present in that molecular weight fraction in the wild-type rice (Hayashi et al., 2018). When the SSI levels are reduced, the molecular weight distribution patterns of the starch biosynthetic enzymes are identical to those in the wild-type japonica rice. However, when SSIIIa is absent and SSI levels are reduced, SSIIa is found only in the 200–300-kDa fraction; by contrast, in the wild-type japonica rice, SSIIa is found in complexes of a wide range of molecular weight (Hayashi et al., 2018). In the absence of SSIIa, the amount of SSI in the 200–300-kDa fraction increases (Miura et al., 2018). Therefore, alterations of the levels of SS isozymes involved in protein complex formation are likely compensated by other SS isozymes in the rice endosperm.

In the absence of SSI, the SSIIa and BEIIb ratio in the 200–300-kDa fraction, in which the trimeric protein complex elutes, to the amount of enzymes in the high-molecular-weight fractions (>700 kDa) was lower than in the wild-type (Figure 2). In addition, the amounts of SSIIa, SSIVb, BEI, BEIIb, and PUL in the high-molecular-weight fractions (fractions 2–4; >700 kDa) were considerably higher than in the wild-type Nip (Figure 2). Similarly, in a leaky *ss1* rice mutant expressing ca. 20% of SSI of that in the wild-type, the amounts of SSIIa, SSIVb, BEI, BEIIb, and PUL eluting in the high-molecular-weight fractions (>700 kDa) were slightly elevated compared to the wild-type (Hayashi et al., 2018), although the degree of increase was lower than that in the *ss1* null mutant analyzed in the current study. Therefore, the amount of SSI may control the amount of the SSI–SSIIa–BEIIb trimeric protein complex, and the absence of SSI alters the interactions during elution of other starch biosynthetic enzymes, particularly SSIIa, SSIVb, BEI, BEIIb, and PUL, which all eluted in high-molecular weight fractions (Figure 2).

Schematic representation of the proposed starch biosynthetic protein complexes in the *ss1* mutant rice (Figure 6B) envisages the recruitment of SSIIa, SSIVb, BEI, BEIIb, and PUL to the high-molecular-weight protein complexes. It may be speculated that the SSI–SSIIa–BEIIb interactions may have priority over the interactions between other starch biosynthetic enzymes, such as BEI–BEIIb and BEI–PUL, in the presence of SSI in the wild-type plant. Therefore, in the absence of SSI, alternative protein complexes may form; however, such alternative protein complexes may be less stable under the experimental conditions used herein since the interactions of SSIIa with other starch biosynthetic enzymes were weakened, as observed by co-immunoprecipitation (Figures 4A,B).

### Loss of BEIIb Activity Alters the Elution Pattern of BEIIa in the Developing Rice Seed

The absence of BEIIb activity drastically affected the elution pattern of BEIIa regardless of the presence or absence of inactive BEIIb. BEIIa eluted in the high-molecular-weight fractions (fractions 2–7), in which no BEIIa was detected in the parental wild-type (Figures 3A,B), while the amount of monomeric BEIIa (fractions 10–13) was reduced compared to the parental wild-type.

By contrast, the pleiotropic effects of other starch biosynthetic enzymes on the elution pattern in the presence or absence of inactive BEIIb protein were different (Figures 3A–C). In the presence of inactive BEIIb protein in line #*1411*, majority of the inactive BEIIb eluted in high-molecular-weight fractions (and was enriched in fractions 2–3), together with SSI, SSIIa, SSIIIa, SSIVb, BEI, and BEIIa (Figures 3A,B). Interactions of inactive BEIIb with SSI, SSIIa, SSIVb, BEI, and BEIIa were indeed observed by reciprocal co-immunoprecipitation, suggesting that all these enzymes may associate with each other to form high-molecular-weight protein complexes, as depicted in Figure 6C.

In the absence of BEIIb, in line *EM10*, the amount of SSI in fractions 4 and 5 was elevated, while that of SSIIa and BEI in fractions 2–6 was lower than in the wild-type Kin (Figures 3B,C). This was partly supported by co-immunoprecipitation analysis, which indicated that the associations SSI–SSIIa, SSI–BEI, SSIIa–BEI, and BEIIa–BEI were weaker than in the wild-type. Therefore, the loss of BEIIb may either reduce the formation or stability of the protein complex under the experimental conditions used (Figures 4A,B), as shown schematically in Figure 6D.

The proposed protein complexes in lines #*1411* and *EM10* are shown in Figures 6C,D, respectively. In the absence of BEIIb activity, BEIIa may form the SSI–SSIIa–BEIIa trimeric protein complex; further, BEIIa may be included in other high-molecular-weight protein complexes (>700 kDa) (Figures 6C,D). The possibility that the high-molecular-weight complexes containing inactive BEIIb protein are associated through long glucan chains cannot be excluded; however, this is unlike since both lines *EM10* and #*1411* produces long-branched amylopectin (Figure 1). Therefore, substitution of Gly645Arg in BEIIb may possibly enhance the association with other starch biosynthetic enzymes.

Line #*1411* and its parental mutant line *ssg3* exhibits flattened seed phenotype compared with line *EM10*, although the chain-length distribution patterns of amylopectin in lines #*1411* and *EM10* are essentially identical (Figure 1). Therefore, the formation of a large protein complex (>700 kDa) containing inactive BEIIb may hinder efficient starch synthesis. In addition, formation of the trimeric protein complex with SSI–SSIIa–BEIIb, not SSI–SSIIa–BEIIa or SSI–SSIIa–inactive BEIIb, may be important for the synthesis of wild-type-like amylopectin structure.

Although the functionality of BEIIb, in terms of generating short amylopectin branches (DP 6–7), cannot be compensated by either BEI or BEIIa (Nishi et al., 2001), the observations made in the current study suggested that BEIIa likely complements the role of BEIIb, to form an alternative protein complex to maintain starch production. The absence of BEIIa alone does not affect the chain length distribution of amylopectin in rice (Nakamura, 2002; Satoh et al., 2003); however, the importance of BEIIa becomes apparent in the absence of BEIIb activity. Further analyses of the possible interacting partners of BEIIa will aid the understanding of the role of BEIIa in protein complex formation.

### Comparisons of the Effect of *be2b* Mutation on Protein Complex Formation of Biosynthetic Starch Enzymes in Rice and Maize

The absence of BEIIb activity results in the production of longer amylopectin branches with reduced branching frequency in both maize kernel (Klucinec and Thompson, 2002; Li et al., 2011) and rice seed (Nishi et al., 2001; Matsushima et al., 2010; Figure 1). However, the effects of absence of BEIIb activity on protein complex formation of starch biosynthetic isozymes in the *be2b* mutants of maize and rice are different. The elution pattern of maize BEI resembles that of BEIIa in rice. BEI in wild-type maize elutes only in fractions smaller than 158 kDa, whereas in maize *be2b* mutant lacking BEIIb protein, BEI elutes in a broad molecular weight range (up to 700 kDa) (Liu et al., 2009). On the other hand, in wild-type japonica rice, BEIIa eluted in a fraction smaller than 300 kDa, while in the rice *be2b* mutants, it eluted in a broad molecular weight range (60–700 kDa) regardless of the presence and absence of inactive BEIIb (Figure 3A). The elution pattern of BEIIa in maize was the same in wild-type and *be2b* null mutant (Hennen-Bierwagen et al., 2009).

The effects of the presence and absence of inactive BEIIb in rice (Figures 3, 4) and maize (Liu et al., 2011) were also different. As described above, in rice, in the presence of inactive BEIIb harboring the Gly645Arg substitution, the inactive BEIIb protein interacted with SSI, SSIIa, SSIVb, BEI, and BEIIa. By contrast, in maize *be2b* mutant that expresses inactive BEIIb protein lacking amino acids 272–299, no increase in its association with other starch biosynthetic enzymes was apparent (Liu et al., 2011). These differences may depend on the nature of the affected primary sequence site. The manner in which the formation of starch biosynthetic protein complexes is compensated differs depending on the presence or absence of each enzyme, and also on the location of amino acid substitutions within the enzymes. Accumulation of such comparative data should reveal protein domains that are important for protein association with starch granules as well as interaction with other enzymes such as kinases and phosphatases, which control the formation of protein complexes.

In maize, the presence and absence of inactive BEIIb in *be2b* mutants resulted in different starch properties, although both *be2b* mutants showed a similar amylopectin branch structures with reduced amylopectin short chains (DP < 15) and increased amylopectin long chains (DP > 16) (Liu et al., 2011). When BEIIb protein was absent in line *ae 1.1*, the size of starch granule was 75% of wild-type and the starch content was 4% less, but it accumulated 2.6-fold more amylose (66%) than wild-type (25%) (Liu et al., 2011). On the other hand, when inactive BEIIb protein was present in line *ae 1.2.*, the size of starch granule was decreased to 50% of wild-type and the starch content was 5% less, but it accumulated only twofold more amylose (49%) than wild-type (Liu et al., 2011). In addition, peak gelatinization temperature was higher in the absence of BEIIb (80°C) compared with the line expressing inactive BEIIb (78°C) due to slight differences in DP16-20. It was concluded that these differences in starch phenotype between the two *be2b* mutants were explained by alterations in protein–protein interactions between enzymes of amylopectin synthesis (Liu et al., 2011).

In this study, the chain length distribution patterns of amylopectin in two *be2b* mutant rice lines, *EM10* and *#1411*, were essentially identical (Figure 1). However, great differences were observed in protein–protein interactions between enzymes of amylopectin synthesis (Figures 3, 4). Therefore, formation of the trimeric protein complex with SSI–SSIIa–BEIIb, not SSI–SSIIa–BEIIa or SSI–SSIIa–inactive BEIIb, may be important for the synthesis of wild-type–like amylopectin structure. The possibility remains that alterations in protein–protein interactions between enzymes of amylopectin synthesis may affect other starch properties such as starch granule morphology, molecular size of whole amylopectin, amylose content, gelatinization temperature, and resistant starch content, in addition to the seed weight described above. The effect of altered protein–protein interaction on these parameters should be examined using back-crossed lines to eliminate the effect of environmental factors.

### Starch Granule-Bound Proteins

Some of the starch biosynthetic proteins are entrapped within starch granules, and can only be extracted when starch is gelatinized (Denyer et al., 1993; Grimaud et al., 2008; Liu et al., 2009; Butardo et al., 2012; Koziol et al., 2012; Cao et al., 2015; Luo et al., 2015; Xing et al., 2016). The composition of starch granule-bound proteins differs among cultivars, mutants, and plant species. However, such granule-bound proteins often contain SSI, SSIIa, and BEIIb, in addition to the granule-bound starch synthase I (GBSSI), which is responsible for amylose synthesis (Denyer et al., 1993; Umemoto and Aoki, 2005; Grimaud et al., 2008; Butardo et al., 2012; Abe et al., 2014; Asai et al., 2014; Luo et al., 2015; Xing et al., 2016; Itoh et al., 2017). The composition of starch granule-bound proteins is thought to reflect the composition of the starch biosynthetic protein complex (Liu et al., 2009, 2011, 2012; Luo et al., 2015). In maize, in the absence of BEIIb protein, BEI and BEIIa are entrapped in the starch granules. In addition, Pho1 is bound to the starch granules in the absence of BEIIb, but not in the presence of inactive BEIIb (Liu et al., 2011). In rice, in the absence of BEIIb protein, SSI, SSIIa, SSIIIa, BEI, and BEIIa are entrapped in the starch granules, as demonstrated by western blotting of starch granule-bound proteins (Itoh et al., 2017); however, whether any other proteins also bind to starch granules remains unknown.

The current study revealed the differences in the composition of starch granule-bound proteins in the *ss1* and *be2b* rice mutants (Figure 5). Three major protein bands were apparent in the three wild-type rice lines, and represented SSI, BEIIb, and GBSSI, as suggested by western blotting (Figures 5A,B). These three proteins also associate with starch granules in wild-type maize (Grimaud et al., 2008; Liu et al., 2009, 2011, 2012), wheat (Peng et al., 2002), and barley (Li et al., 2011). One of the bands, which migrated as an approximately 70-kDa protein in wild-type japonica rice Nip was absent in the *ss1* mutant; hence, this protein was likely SSI (Figures 5A,B). Only a small amount of SSIIa was associated with starch granules in the *ss1* mutant and wild-type japonica rice Nip, Kin, and Akt (Figure 5B). This may be because of two amino acid substitutions, Glu88Asp and Gly604Ser, commonly found in the low-activity type SSIIa in typical japonica rice (Nakamura et al., 2005). Active SSIIa from typical indica rice can indeed associate with starch granules (Nakamura et al., 2005; Bao et al., 2009).

In the current study, the number and amounts of starch granule-bound proteins detected in both *be2b* mutant lines were greater than those in their parental wild-types. Further, the patterns of starch granule-bound proteins were similar in the two *be2b* mutants, regardless of the presence (line #*1411*) or absence (line *EM10*) of inactive BEIIb protein. The only exception was the presence of small amounts of BEIIb in line #*1411* (Figures 5A,B). The starch granule-bound proteins from line *EM10* were identified by nano-LC-MS/MS, and included SSIIIa, Pho1, PUL, PPDK, BEIIa, SSIIa, BEI, heat shock proteins 70, and SSI (Figure 5A and Table 1). In addition, the presence of GBSSI was indicated by western blotting (Figure 5B). Starch granule-bound proteins from the *be2b* mutant of maize similarly contain SSIIIa, Pho1, BEIIa, SSIIa, BEI, and SSI (Grimaud et al., 2008; Liu et al., 2009). Association of these starch biosynthetic proteins with the starch granule in the absence of BEIIb activity is conserved in japonica rice and maize, although the association of Pho1 with the starch granule differs depending on the affected site in the BEIIb protein in maize (Liu et al., 2009, 2011).

Starch biosynthetic enzymes that form a protein complex in maize stroma were suggested to bind to starch granules therein (Liu et al., 2009, 2011, 2012). However, the observation made in the course of the present study suggested that the binding of starch biosynthetic enzymes to starch granules might also depend on the amylopectin structure as both indica rice and *be2b* mutant rice lines used this study show less short chains with DP 8–12 and more long chains (Nishi et al., 2001; Umemoto et al., 2002).

Starch granule-bound Pho1, BEI, and BEIIb are phosphorylated in maize (Liu et al., 2009, 2011), and the phosphorylation site in BEIIb has been identified (Makhmoudova et al., 2014). However, the major phosphorylation site in maize BEIIb is not conserved in rice (Crofts et al., 2017b). Therefore, it is of interest to investigate whether these proteins have alterative phosphorylation sites. Current study used soluble proteins extracted from the developing rice seeds. Therefore, *in vitro* reconstitution and crystallization of specific protein complexes will provide further insight into the mechanisms responsible for starch synthesis.

## Author Contributions

NC, YI, NA, SM, and KK performed the experiments. NC wrote the manuscript. NF conceived the original research plans and supervised the research. NF and RM contributed to the manuscript writing.

## Conflict of Interest Statement

The authors declare that the research was conducted in the absence of any commercial or financial relationships that could be construed as a potential conflict of interest. The handling Editor declared a past co-authorship with the authors NC and NF and a shared affiliation, though no other collaboration, with the authors NC, YI, NA, SM, RM, and NF.
