# Supplementary material for: Rice Mutants Lacking Starch Synthase I or Branching Enzyme IIb Activity Altered Starch Biosynthetic Protein Complexes
Source: Front Plant Sci. 2018 Dec 7;9:1817. doi: 10.3389/fpls.2018.01817 (PMC6292963; doi:10.3389/fpls.2018.01817)
Supplement: Supplementary file 1 [file Data_Sheet_1.pdf]

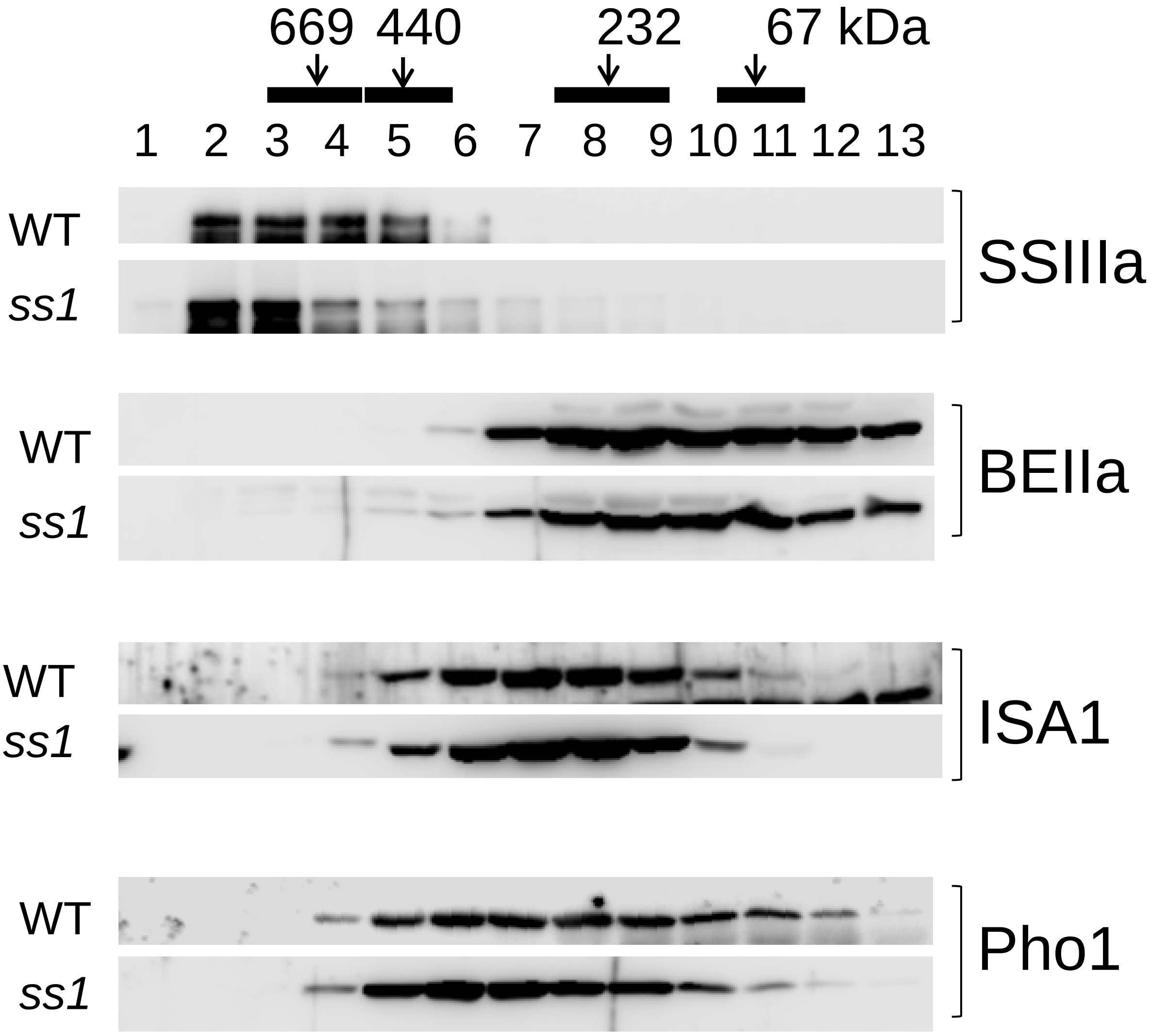

SUPPLEMENTARY FIGURE 1| Molecular weight distributions of SSIIIa, BEIa, ISA1, and Pho1 in the *ss1* mutant and its parental line, Nip. Fractions 1–13 were obtained after gel filtration chromatography of soluble proteins extracted from the developing seeds and were analyzed by western blotting with the indicated antibodies. Top, numbers (kDa) indicate molecular weight standards. The images are representative of three.

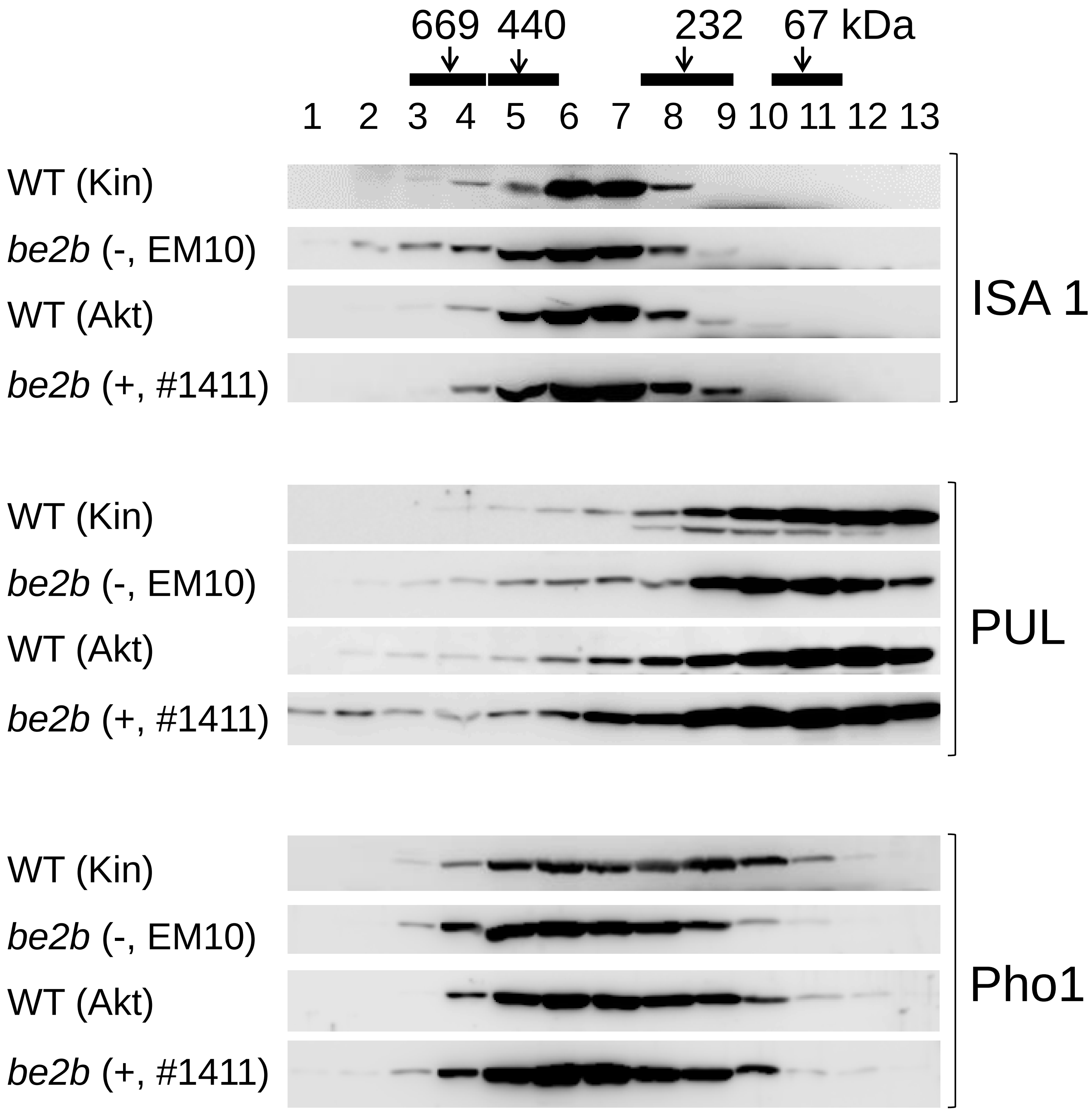

SUPPLEMENTARY FIGURE 2| Molecular weight distributions of ISA1, PUL, and Pho1 in the *be2b* mutants and their parental lines. Fractions 1–13 were obtained after gel filtration chromatography of soluble proteins extracted from developing seeds and were analyzed by western blotting with the indicated antibodies. Top, numbers (kDa) indicate molecular weight standards. The images are representative of three.

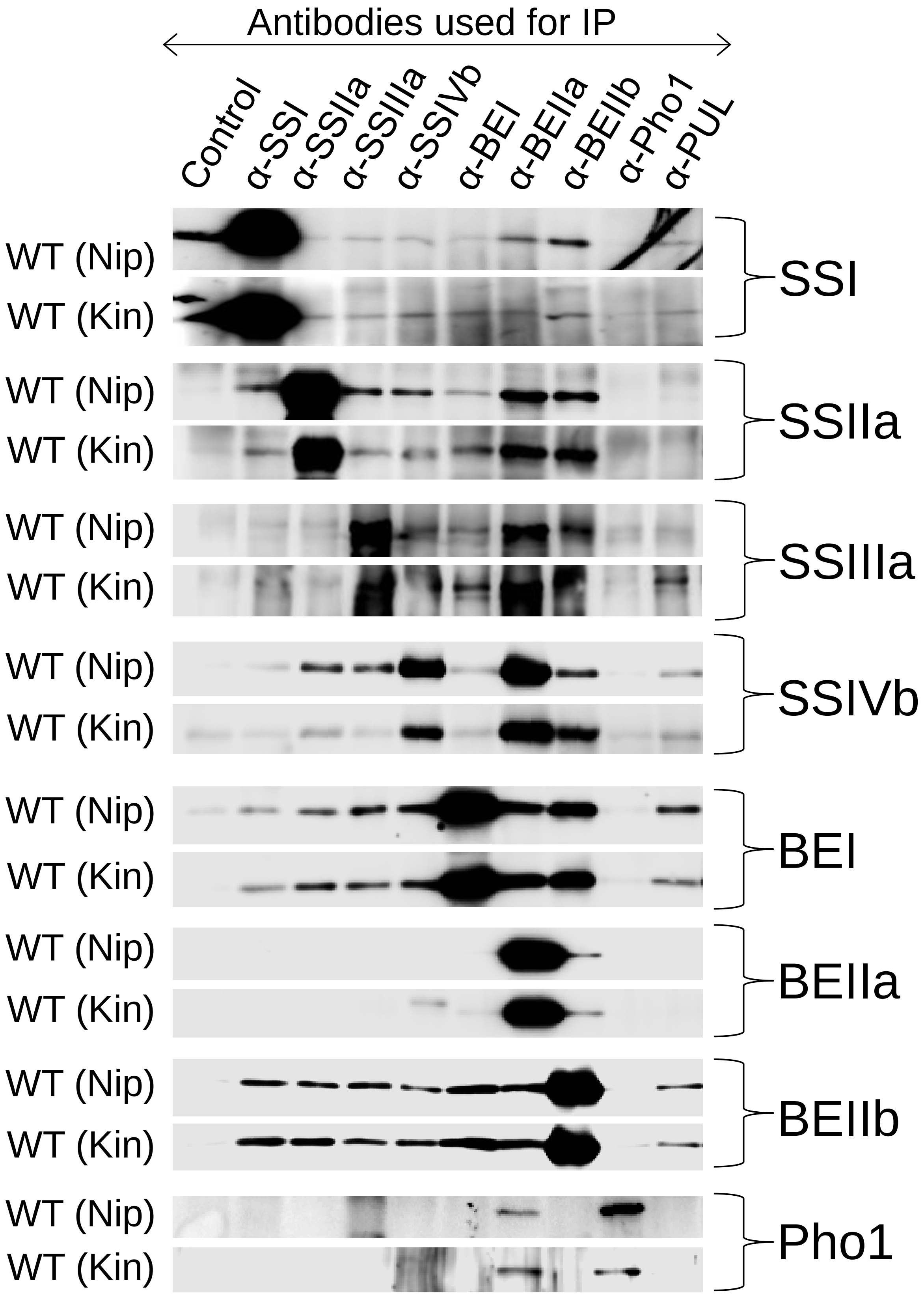

Supplementary Figure 3A

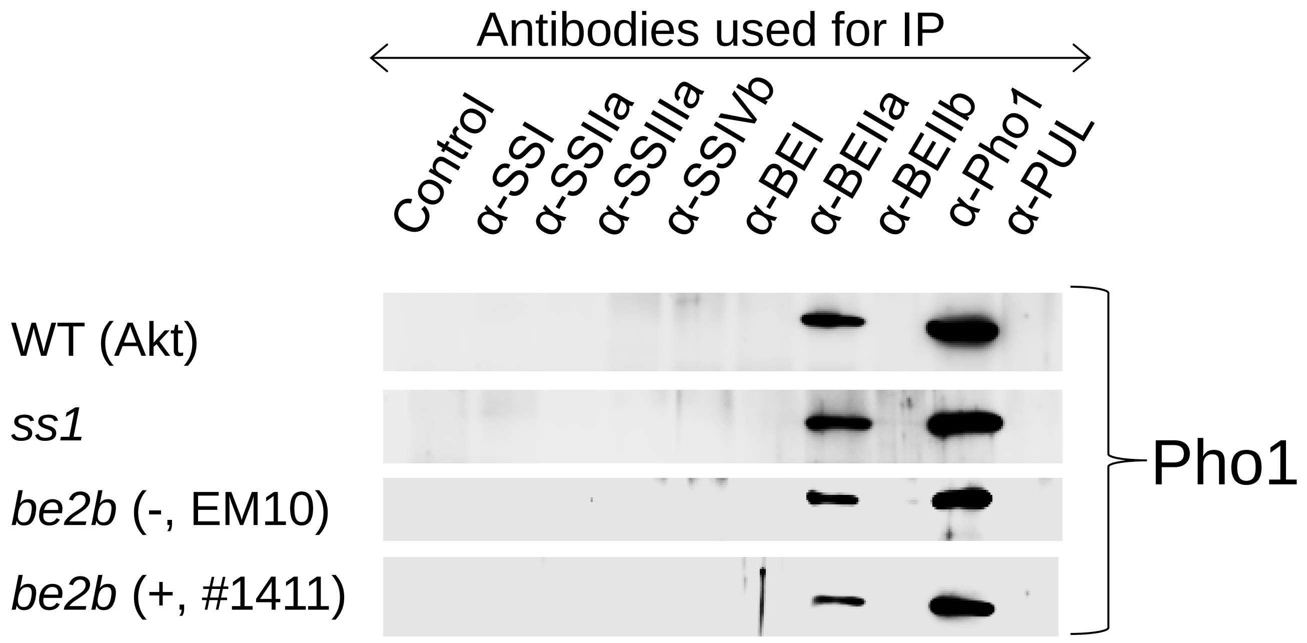

Supplementary Figure 3B

SUPPLEMENTARY FIGURE 3| Co-immunoprecipitation of proteins from the *ss1* and *be2b* mutants, and their parental lines. Soluble proteins extracted from the developing seeds were incubated with isozyme-specific antibodies, or pre-immune sera as a control (indicated at the top), and protein A sepharose. After washing, the captured proteins were analyzed by western blotting with antibodies indicated on the right. **(A)** Co-immunoprecipitation of proteins from the wild types, Nip and Kin. **(B)** Western blotting of immunoprecipitated material from the *ss1* and *be2b* mutants (lines *EM10* and *#1411*), and wild type (Akt) using anti-Pho1 antibody. The images are representative of at least three.

Starch synthase IIIa (SSIIIa)
gi|40253646

**1**	MEMALRPQSL	LCPRSRLKVV	IRPASSASGG	GLAQYFLMTR	RYTGSRIVRC
**51**	MVSSSDCPNR	KAKRTISLHT	EVASSRGYAP	R**IAAESSIQE**	**R**EHINSDEET
**101**	FDTYNRLLRN	ESTEWK**KLDT**	**TEVDLSQDVS**	**SSSMR**KVDAT	DEAK**LDILED**
**151**	**DLPR**NLLNGV	TMGEVDMLDE	AGAEDDVFEV	DLSALHNSTV	GK**MDAVNEVG**
**201**	**TENDLFEVDL**	**SALHSAAVGK**	VDVVDGAK**AK**	**EDLFEMDSLA**	**LHSVTMGK**VD
**251**	AINAAGAEGD	KFEVDLSALA	SNNSMIEAVN	VMDEAKAIED	TLEVDLSGNA
**301**	TSSSTYGEVK	FEVDSLGNTS	STVMYGPADG	AYEPRSDEVT	FKVDSSENAS
**351**	NNVMYGRADV	VDESWADEGI	FEVDFFTNAS	SGAEYGKVDV	VDEAK**TDDFT**
**401**	**FEIDSLEK**DS	NNKMHGKAHM	VDEAWDDEAI	FEVDLFGNAS	SIPIYGEVNV
**451**	LDEARADDGK	FEVDLLGNTS	SNSTHEEVDV	VDEAQTGEAT	FEVDLLGNAL
**501**	SSAIYKEVPV	MGGAQDDEVD	VDFSINASIT	ETEKEADAVD	EARVEDETFD
**551**	MDLVGK**QISI**	**DSMNDDVVEE**	**GTK**HHR**YPML**	**SSAFIEVK**TI	HETPVSLKPE
**601**	LMSVVMDQEQ	DKPISSVYQQ	EGSIFNLHAE	NQSTVDFHER	**EQMAITFDK**Q
**651**	KESVAKLSKE	DQQTAGLPEQ	NMSFDGVHRK	**SQSIIGLPFQ**	**HQSIVSSPEK**
**701**	YR**SIVGFHGQ**	**NQSIISSHKQ**	**DK**SIVGVPKK	IQSIVGSTK**H**	**DDSIVGFR**KQ
**751**	DR**SIVSVPEQ**	**KQSIVGFHK**Q	DLSIVAVSEQ	NLSIVAIPRE	SQSKQISIVR
**801**	RHDPLHLKEV	ETKDRDGISK	KSGGDDDLPH	MLFEEELSQV	EDVARAIAYK
**851**	KQHEVDVISL	TPDIQESPQD	NIDPQELRRM	LQELADQNCS	MGNK**LFVFPE**
**901**	**AVKANSTIDV**	**YLNRNLSALA**	**NEPDVHIK**GA	FNSWRWRPFT	ERLHKSELSG
**951**	DWWSCKLHIP	KEAYRLDFVF	FNGRLVYDNN	DSNDFVLQVE	STMDEDSFEE
**1001**	FLVEEKKREL	ERVATEEAER	RRHAEEQQRM	GEQRAAEQAA	REQAKKEIEL
**1051**	KKNK**LQNLLS**	**SARTHVDNLW**	**HIEPSTYR**QG	DTVRLYYNRN	SRPLMHSTEI
**1101**	WMHGGCNSWT	DGLSIVERLV	ECDDENGDWW	YANVHIPEK**A**	**FVLDWVFADG**
**1151**	**PPGNAR**NYDN	NGRQDFHAIL	PNAMTNEEYW	VEEENCIYTR	LLHEIREREE
**1201**	AIKIKVEKRA	KMKSEMKEKT	MR**MFLLSQKH**	**IVYTEPLEIR**	**AGTTVDVLYN**
**1251**	**PSNTVLNGKP**	**EVWFR**WSFNR	WMHPSGVLPP	KKMVKTEDGC	HLKATVSVPS
**1301**	DAYMMDFVFS	ESEEGGIYDN	R**NGTDYHIPV**	**SGSNAKEPPI**	**HIVHIAVEMA**
**1351**	**PIAKVGGLAD**	**VVTSLSRAIQ**	**ELGHHVEVIL**	**PKYNFMNQSN**	**VK**NLHVR**QSF**
**1401**	**SLGGTEIK**VW	FGLVEDLSVY	FLEPQNGMFG	GGWVYGGNDA	GRFGLFCQSA
**1451**	LEFLLQSGSS	PHIIHCHDWS	SAPVAWLYKE	HYAESRLATA	R**IIFTIHNLE**
**1501**	**FGAHFIGK**AM	TYCDKATTVS	HTYSKEVAGH	GAIAPHRGKF	YGILNGIDPD
**1551**	IWDPYTDNFI	PMHYTSENVV	EGKNAAKRAL	QQR**FGLQQTD**	**VPIVGIITR**L
**1601**	TAQKGIHLIK	HALHRTLER**N**	**GQVVLLGSAP**	**DPR**IQSDFCR	LADSLHGENH
**1651**	GRVRLCLTYD	EPLSHLIYAG	SDFILVPSIF	EPCGLTQLVA	MRYGSIPIVR
**1701**	KTGGLYDTVF	DVDHDKDRAR	VLGLEPNGFS	FDGADCNGVD	YALNRQR**AIS**
**1751**	**SWFEAR**GWFH	SLCKRVMEQD	WSWNRPALDY	IELYHSAHKF	

SUPPLEMENTARY FIGURE 4A| SSIIIa identified by nano-LC-MS/MS in starch granule-bound protein of line *EM10*. The identified peptides are indicated in red.

Plastidial starch phosphorylase 1 (Pho1) gi|190689248

1 MATASAPLQL ATASRPLPVG VGCGGGGGGG LHVGGARGGG AAPARRRLAV
51 RSVASDR**GVQ** **GSVSPEEEIS** **SVLNSIDSST** **IASNIKHHAE** **FTPVFSPEHF**
101 **SPLK**AYHATA KSVLDTLIMN WNATYDYYDR TNVK**QAYYLS** **MEFLQGR**ALT
151 NAVGNLELTG QYAEALQQLG HSLEDVATQE PDAALGNGGL GRLASCFLDS
201 LATLNYPAWG YGLRYKHGLF KQIITKDGQE EVAENWLEMG NPWEIVRTDV
251 SYPVKFYGKV VEGTDGRMHW IGGENIK**VVA** **HDIPIPGYK**T KTTNNLR**LWS**
301 **TTVPSQDFDL** **EAFNAGDHAS** **AYEAHLNAEK** **ICHVLYPGDE** **SPEGK**VLR**LK**
351 **QQYTLCSASL** **QDIIAR**FERR **AGDSLSWEDF** **PSKVAVQMND** **THPTLCIPEL**
401 **MR**ILIDVKGL SWNEAWSITE R**TVAYTNHTV** **LPEALEK**WSL DIMQKLLPR**H**
451 **VEIIEKIDGE** **LMNIIISKYG** **TEDTSLLKK**K IKEMR**ILDNI** **DLPDSIAK**LF
501 VKPKEKKESP AKLKEKLLVK **SLEPSVVVEE** **K**TVSKVEINE DSEEVEVDSE
551 EVVEAENEDS EDELDPFVKS DPKLPRVVRM ANLCVVGGHS VNGVAAIHSE
601 IVKEDVFNSF YEMWPAKFQN KTNGVTPRRW IR**FCNPELSA** **IISK**WIGSDD
651 WVLNTDKLAE LKKFADDEDL QSEWRAAKKA NK**VKVVSLIR** EK**TGYIVSPD**
701 **AMFDVQVK**RI HEYKR**QLLNI** **LGIVYR**YKKM KEMSAK**DRIN** **SFVPR**VCIFG
751 GK**AFATYVQA** **K**RIVK**FITDV** **AATVNHDPEI** **GDLLK**VVFIP DYNVSVAEAL
801 IPASELSQHI STAGMEASGT SNMKFAMNGC ILIGTLDGAN VEIREEVGEE
851 NFFLFGAEAH EIAGLRKERA QGKFVPDPRF EEVKRFVRSG VFGTYNYDDL
901 MGSLEGNEGY GR**ADYFLVGK** **DFPSYIECQE** **K**VDKAYRDQK LWTR**MSILNT**
951 **ASSSK**FNSDR TIHEYAK**DIW** **DIKPVILP**

SUPPLEMENTARY FIGURE 4B| Pho1 identified by nano-LC-MS/MS in starch granule-bound protein of line *EM10*. The identified peptides are indicated in red.

Pullulanase (PUL)
gi|222628355

1 M**AVGEECAAA VASQGFVTDA R**AYWVTR**SLI AWNVNDQDTS LFLYASRDAT**

51 **MHVSDGAIHG YDSKIELEPE HASLPDNVAE K**FPFIRSYR**T FRVPSSVDVA**

101 **SLVKCQLAVA SYDAHGR**HQD VTGLQLPGVL DDMFAYTGPL GAVFSDKDVD

151 LYLWAPTAQD VR**VCFYDGPA GPLLQTVQLK ELNGVWSVTV PRYRENQYYL**

201 **YEVKVYHPST SQVEKCLADD PYAR**GLSANG TRTWLVDINS ETLKPASWDE

251 LSDEKPNLES FSDISIYELH IR**DFSAHDST VDCNSR**GGFR **AFTFQDSAGI**

301 **R**HLR**KLSAAG LTHVHLLPSF HFASVDDNK**S NWK**FVDEAQL AKLPPGSDEQ**

351 **QAAIVSIQQE DPYNWGYDPV LWGVPKGSYA SNPDGPSR**II EYR**QMVQALN**

401 **R**IGLR**VVMDV VYNHLDSSGP FGVSSVLDKI VPGYYLR**R**NV NGQIENSAAM**

451 **NNTASEHFMV DRLIVDDLLN WAINYK**VDGF R**FDLMGHIMK** STMIRAKSAI

501 RSLTRDVHGV DGSK**IYLYGE GWDFGEVAQN KRGINASQIN MSGTGIGSFN**

551 **DR**IRDSVNGG NPFGNPLQQG FSTGLFLEPN GYYQGNEADT RR**ELATYADH**

601 **IQIGLAGNLK DYVLR**THTGE AKKGSDIYTF DGSPVGYTSS PVETINYVSA

651 HDNETLFDIV SIK**TPIGLSI DEK**CR**INHLA SSMIALSQGI PFFHAGDEIL**

701 **R**SKSLDRDSY NSGDWFNKLD FTYETNNWGV GLPPR**DKNEE NWHLIKPRLE**

751 **NPSFRPLKNH ILSVFDNFVD ILK**IR**YSSPL FRLSTASDIE QR**VR**FHNTGP**

801 **SMVPGVIVMS IK**DAQNEKCK MAQLDKNFSY VVTIFNVCPH EVSIEIHDLA

851 SLGLELHPIQ VNSSDALVRQ SAYEASK**GRF TVPRRTTAVF VQPR**C

SUPPLEMENTARY FIGURE 4C| PUL identified by nano-LC-MS/MS in starch granule-bound protein of line *EM10*. The identified peptides are indicated in red.

Pyruvate phosphate dikinase 1 (PPDK)
gi|218196777

1 MAPAQCARVQ RVFHFGKGKS EGNKAMKDLA CQQYQAAGKT LPAGLWEEIV

51 EGLQWVEEYM AAR**LGDPARP LLLSVR**SGAA VSMPGMMDTV LNLGLNDEVA

101 AGLAAKSGDR FAYDSYRRFL DMFGNVVMDI PHALFEEKLE AMKAVK**GLHN**

151 **DTDLTATDLK** ELVAQYKDVY VEAK**GEPFPS DPKK**QLQLAV LAVFNSWDSP

201 RAIKYRSINK ITGLKGTAVN VQTMVFGNMG NTSGTGVLFT RNPSTGEKKL

251 YGEFLVNAQG EDVVAGIRTP EDLDAMRDHM PEPYEELVEN CKILESHYKE

301 MMDIEFTVQE NRLWMLQCRT GKRTGKGAVK **IAVDMVNEGL VER**RTALKMV

351 EPGHLDQLLH PQFENPSGYK DKVIATGLPA SPGAAVGQIV FTAEDAEAWH

401 AQGKDVILVR **TETSPEDVGG MHAAVGILTA R**GGMTSHAAV VARGWGKCCV

451 SGCSSVRVND ASK**IVVIEDK** ALHEGEWLSL NGSTGEVIIG KQPLCPPALS

501 GDLETFMSWV DEVRK**LKVMA NADTPEDATT ARQNGAEGIG LCR**TEHMFFA

551 SDERIKAVR**Q MIMASSLELR** QKALDRLLPY QRSDFEGIFR **AMDGLPVTIR**

601 **LLDPPLHEFL PEGHVEDMVR ELCSETGAAQ DDVLAR**VEK**L SEVNPMLGFR**

651 GCR**LGISYPE LTEMQAR**AIF EAAITMTNQG IQVFPEIMVP LVGTPQELGH

701 QVDVIRQIAN KVFTDMGKTI GYK**VGTMIEI PR**AALVADEI AEQAEFFSFG

751 TNDLTQMTFG YSRDDVGKFL PIYLSQGILQ HDPFEVLDQR GVGELVKLAT

801 ERGRKARPNL KVGICGEHGG EPLSVAFFAK AGLDYVSCSP FRVPIARLAA

851 AQVLL

SUPPLEMENTARY FIGURE 4D| PPDK identified by nano-LC-MS/MS in starch granule-bound protein of line *EM10*. The identified peptides are indicated in red.

Branching enzyme IIa (BEIIa)
gi|5689138

1 MASFAVSGAR LGVVRAGGGG GGGGGPAARS GGVDLPSVLF RRKDSFSRGV

51 VSCAGAPGKV LVPGGGSDDL LSSAEPDVET QEQPEESQIP DDNK**VKPFEE**

101 **EEEIPAVAEA SIK**VVAEDKL ESSEVIQDIE ENVTEGVIKD ADEPTVEDKP

151 R**VIPPPGDGQ KIYQIDPMLE GFR**NHLDYRY SEYKRMR**AAI DQHEGGLDAF**

201 **SR**GYEKLGFT R**SAEGITYR**E WAPGAQSAAL VGDFNNWNPN ADTMTR**NEYG**

251 **VWEISLPNNA DGSPAIPHGS R**VKIR**MDTPS GVKDSIPAWI KFAVQAPGEI**

301 **PYNGIYYDPP EEEKYVFQHP QPK**RPNSLR**I YESHIGMSSP EPKINTYANF**

351 **RDEVLPR**IKK LGYNAVQIMA IQEHSYYASF GYHVTNFFAP SSR**FGTPEDL**

401 **K**SLIDKAHEL GLLVLMDIVH SHASNNTLDG LNGFDGTDTH YFHGGPRGHH

451 WMWDSR**LFNY GSWEVLR**YLL SNARWWLEEY KFDGFRFDGV TSMMYTHHGL

501 QVAFTGNYGE YFGFATDVDA VVYLMLVNDL IHGLYPEAVA IGEDVSGMPT

551 FCIPVQDGGV GFDYR**LHMAV PDK**WIELLKQ SDEYWK**MGDI VHTLTNRR**WS

601 EK**CVTYAESH DQALVGDK**TI AFWLMDKDMY DFMALDRPST PRIDRGIALH

651 KMIRLVTMGL GGEGYLNFMG NEFGHPEWID FPR**GPQSLPN GSVLPGNNYS**

701 **FDK**CRRR**FDL GDADYLRYHG MQEFDQAMQH LEEKYGFMTS EHQYISR**KHE

751 EDKVIIFERG DLVFVFNFHW SNSYFDYRVG CLKPGKYK**IV LDSDDGLFGG**

801 **FSR**LDHDAEY FTADWPHDNR PCSFSVYTPS RTAVVYALTE D

SUPPLEMENTARY FIGURE 4E| BEIIa identified by nano-LC-MS/MS in starch granule-bound protein of line *EM10*. The identified peptides are indicated in red.

Starch synthase IIa (SSIIa)
gi|60417785

1 MSSAVVASST TFLVALASSA SRGGPRRGRV VGVAAPPALL YDGRAGRLAL

51 RAPPPPRPRP RRRDAGVVRR **ADDGENEAAV ERAGEDDDEE EEFSSGAWQP**

101 **PR**SRRGGVGK VLKRRGTVPP VGRYGSGGDA AR**VRGAAAPA PAPTQDAASS**

151 **KNGALLSGRD DDTPASRNGS VVTGADKPAA ATPPVTITK**L PAPDSPVILP

201 SVDKPQPEFV IPDATAPAPP PPGSNPR**SSA PLPKPDNSEF AEDKSAK**VVE

251 SAPKPKATR**S SPIPAVEEET WDFKK**YFDLN EPDAAEDGDD DDDWADSDAS

301 DSEIDQDDDS GPLAGENVMN VIVVAAECSP WCK**TGGLGDV AGALPK**ALAR

351 RGHR**VMVVVP RYGDYAEAQD VGIRK**YYK**AA GQDLEVK**YFH AFIDGVDFVF

401 IDAPLFRHRQ DDIYGGNRQE IMKR**MILFCK** AAVEVPWHVP CGGVPYGDGN

451 LVFLANDWHT ALLPVYLKAY YRDNGMMQYT R**SVLVIHNIA YQGRGPVDEF**

501 **PYMELPEHYL DHFKLYDPVG GEHANIFGAG LK**MADR**VVTV SPGYLWELKT**

551 **TEGGWGLHDI IR**ENDWK**MNG IVNGIDYREW NPEVDVHLQS DGYANYTVAS**

601 **LDSSKPR**CKA ALQR**ELGLEV RDDVPLIGFI GR**LDGQK**GVD IIGDAMPWIA**

651 **GQDVQLVLLG SGRRDLEVML QR**FEAQHNSK **VRGWVGFSVK** MAHR**ITAGAD**

701 **VLVMPSRFEP CGLNQLYAMA YGTVPVVHAV GGLRDTVSAF DPFEDTGLGW**

751 **TFDR**AEPHK**L IEALGHCLET YRK**YKESWRG FQVR**GMSQDL SWDHAAELYE**

801 **EVLVK**AKYQW

SUPPLEMENTARY FIGURE 4F| SSIIa identified by nano-LC-MS/MS in starch granule-bound protein of line *EM10*. The identified peptides are indicated in red.

Branching enzyme I (BEI)
gi|218149

1 MLCLTSSSSS APPPLLPSLA DRPSPGIAGG GGNVRLSVVS SPRRSWPGKV

51 KTNFSVPATA RK**NKTMVTVV** **EEVDHLPIYD** **LDPKLEEFKD** **HFNYR**IKRYL

101 DQK**CLIEKHE** **GGLEEFSK**GY LK**FGINTVDG** **ATIYR**EWAPA AQEAQLIGEF

151 NNWNGAKHKM EK**DKFGIWSI** **KISHVNGKPA** **IPHNSK**VKFR FRHGGGAWVD

201 RIPAWIRYAT FDASK**FGAPY** **DGVHWDPPAC** **ER**YVFKHPRP PKPDAPR**IYE**

251 **AHVGMSGEEP** **EVSTYREFAD** **NVLPR**IRANN YNTVQLMAIM EHSYYASFGY

301 HVTNFFAVSS R**SGTPEDLKY** **LVDKAHSLGL** **RVLMDVVHSH** **ASNNVTDGLN**

351 **GYDVGQNTHE** **SYFHTGDR**GY HKLWDSR**LFN** **YANWEVLR**FL LSNLRYWMDE

401 FMFDGFR**FDG** **VTSMLYHHHG** **INK**GFTGNYK EYFSLDTDVD AIVYMMLANH

451 LMHK**LLPEAT** **IVAEDVSGMP** **VLCRPVDEGG** **VGFDFRLAMA** **IPDRWIDYLK**

501 NKEDRK**WSMS** **EIVQTLTNRR** YTEK**CIAYAE** **SHDQSIVGDK** **TIAFLLMDK**E

551 MYTGMSDLQP ASPTINRGIA LQKMIHFITM ALGGDGYLNF MGNEFGHPEW

601 IDFPREGNNW SYDKCR**RQWS** **LVDTDHLR**YK YMNAFDQAMN ALEEEFSFLS

651 SSKQIVSDMN EK**DKVIVFER** **GDLVFVFNFH** **PNKTYK**GYK**V** **GCDLPGK**YR**V**

701 **ALDSDALVFG** **GHGRVGHDVD** **HFTSPEGMPG** **VPETNFNNRP** **NSFK**VLSPPR

751 **TCVAYYR**VDE DREELRRGGA VASGKIVTEY IDVEATSGET ISGGWKGSEK

801 DDCGKKGMKF VFRSSDEDCK

SUPPLEMENTARY FIGURE 4G| BEI identified by nano-LC-MS/MS in starch granule-bound protein of line *EM10*. The identified peptides are indicated in red.

70 kDa Heat shock protein
gi|218186646

1 MASFTSQLGA MACGAAPSTS PLAARRSGQL FVGRKPAAAS VQMRVPRAGR

51 ARGVAMRVAC EK**VVGIDLGT TNSAVAAMEG GKPTVITNAE GQRTTPSVVA**

101 **YTK**GGERLVG QIAKR**QAVVN PENTFFSVKR** FIGRKMAEVD DEAK**QVSYHV**

151 **VRDDNGNVKL DCPAIGKQFA AEEISAQVLR K**LVDDASKFL NDKITK**AVVT**

201 **VPAYFNDSQR** TATKDAGR**IA GLEVLRIINE PTAASLAYGF EKK**NNETILV

251 FDLGGGTFDV SVLEVGDGVF EVLSTSGDTH LGGDDFDKFY FCWVFYFGAM

301 THETPK**VVDW LASNFKKDEG IDLLKDK**QAL QRLTEAAEK**A KMELSTLSQT**

351 **NISLPFITAT ADGPKHIETT LSR**AKFEELC SDLIDRLK**TP VTNALR**DAK**L**

401 **SVDNLDEVIL VGGSTRIPSV QELVK**KITGK DPNVTVNPDE VVSLGAAVQG

451 GVLAGDVKDV VLLDVTPLSL GLETLGGVMT KIIPR**NTTLP TSKSEVFSTA**

501 **ADGQTSVEIN VLQGER**EFVR DNKSLGSFR**L DGIPPAPRGV PQIEVKFDID**

551 **ANGILSVAAI DKGTGK**K**QDI TITGASTLPK DEVER**MVEEA DKFAQEDKEK

601 RDAIDTK**NQA DSVVYQTEK**Q LK**ELGDKVPA PVK**EKVDAKL NELKEAIAGG

651 STQSMKDAMA ALNEEVMQIG QAMYNQQPNA GAAGPTPGAD AGPTSSGGK**G**

701 **PNDGDVIDAD FTDSN**

SUPPLEMENTARY FIGURE 4H| 70 kDa Heat shock protein identified by nano-LC-MS/MS in starch granule-bound protein of line *EM10*. The identified peptides are indicated in red.

Starch synthase I (SSI)
gi|122168579

1 MATAAGMGIG AACLVAPQVR PGRRLRLQRV RRRCVAELSR **DGGSAQRPLA**

51 **PAPLVK**QPVL PTFLVPTSTP PAPTQSPAPA PTPPPLPDSG VGEIEPDLEG

101 LTEDSIDK**TI** **FVASEQESEI** **MDVKEQAQAK** VTR**SVVFVTG** **EASPYAKSGG**

151 **LGDVCGSLPI** **ALALR**GHR**VM** **VVMPR**YMNGA LNK**NFANAFY** **TEK**HIK**IPCF**

201 **GGEHEVTFFH** **EYR**DSVDWVF VDHPSYHRPG NLYGDNFGAF GDNQFRYTLL

251 CYAACEAPLI LELGGYIYGQ K**CMFVVNDWH** **ASLVPVLLAA** **KYRPYGVYR**D

301 ARSVLVIHNL AHQGVEPAST YPDLGLPPEW YGALEWVFPE WARR**HALDKG**

351 **EAVNFLK**GAV VTADR**IVTVS** **QGYSWEVTTA** **EGGQGLNELL** **SSR**KSVLNGI

401 VNGIDINDWN PSTDK**FLPYH** **YSVDDLSGK**A KCKAELQK**EL** **GLPIRPDVPL**

451 **IGFIGR**LDYQ KGIDLIK**LAI** **PDLMR**DNIQF VMLGSGDPGF EGWMRSTESG

501 YRDKFR**GWVG** **FSVPVSHRIT** **AGCDILLMPS** **RFEPCGLNQL** **YAMQYGTVPV**

551 **VHGTGGLRDT** **VENFNPFAEK** **GEQGTGWAFS** **PLTIEK**MLWA LR**MAISTYR**E

601 HK**SSWEGLMK** **R**GMSSDFTWD HAASQYEQIF EWAFMDQPYV M

SUPPLEMENTARY FIGURE 4I| SSI identified by nano-LC-MS/MS in starch granule-bound protein of line *EM10*. The identified peptides are indicated in red.
